# Novel Mutations of PAX6 and WFS1 Associated With Congenital Cataract in a Chinese Family

**DOI:** 10.7759/cureus.34208

**Published:** 2023-01-25

**Authors:** Dan Sheng, Duo Yang, Wanqin Xie, Mojiang Li, Liqin Zhong, Shuangxi Zhao, Hao Liang

**Affiliations:** 1 Institute of Traditional Chinese Medicine Diagnostics, Hunan University of Chinese Medicine, Changsha, CHN; 2 Ophthalmology Department, Jili Hospital, Liuyang, CHN; 3 NHC Key Laboratory of Birth Defects for Research and Prevention, Hunan Provincial Maternal and Child Health Care Hospital, Changsha, CHN

**Keywords:** mutation, wfs1, pax6, chinese, congenital cataract

## Abstract

Background: Congenital cataract is a common cause of blindness in childhood. About half of the cases have a genetic etiology, and more than 100 genes have been associated with congenital cataracts. This study reports the clinical and genetic findings of a two-generation Chinese family affected by congenital cataract.

Methods: Ophthalmologic examinations were performed for clinical evaluation of the cataract patients. Whole exome sequencing (WES) and Sanger sequencing were used to identify potentially relevant mutations. The online programsProtein Variation Effect Analyzer (PROVEAN) and Sorting Intolerant from Tolerant (SIFT) were employed to predict the impact of variation on protein function.

Results: Both the proband and her mother were blind because of bilateral nuclear cataracts, and the elder brother of the proband also manifested obvious bilateral cataracts. Sanger sequencing confirmed the mutations in the proband as well as in her mother. The elder brother simply carried the PAX6 c.221G>A variation. The WFS1 c.2070_2079del variation potentially generates a loss-of-function mutant.

Conclusion: The novel PAX6mutation (c.221G>A) is associated with congenital cataract, and the WFS1 mutation (c.2070_2079del) may interactively aggravates this process. These findings may increase our understanding of the genetic etiology of congenital cataract.

## Introduction

Congenital cataract is a common cause of blindness in childhood, with a pooled prevalence of 4.24 per 10,000 people [1]. It is clinically heterogeneous, manifesting different syndromic and nonsyndromic forms. It is morphologically classified into different subtypes, and around 50% of cases are inherited from parents [2,3]. To date, over 100 genes have been reported to be associated with cataracts, which are predominantly of autosomal dominant inheritance [4].

The PAX6 gene (OMIM*607108), located on chromosome 11p13, plays a crucial role in eye development and morphogenesis [5]. It is expressed in the developing iris, lens, ciliary body, corneal epithelium, and retina. To date, more than 400 mutations in the PAX6 have been documented. A large part of patients carrying PAX6 mutations presented with aniridia phenotypes [6]. The congenital cataract has been rarely reported as a cardinal sign of those inherited eye diseases with PAX6 mutations [4]. The WFS1 gene (OMIM*606201) encodes a protein that is thought to regulate calcium homeostasis in cells. Loss-of-function mutations in WFS1 are known to cause autosomal-recessive Wolfram syndrome, a severe neurodegenerative disease mainly characterized by juvenile-onset diabetes mellitus and optic atrophy (OMIM#222300), whereas dominant pathogenic variants in WFS1 were found to cause isolated sensorineural hearing loss (SNHL), syndromic SNHL, congenital cataracts, or early onset diabetes mellitus [7-9].

This study reports the clinical and genetic findings of PAX6 and WFS1 from a two-generation Chinese family affected by congenital cataract.

## Materials and methods

Ethic compliance

The Ethics Committee of Jili Hospital, Liuyang, China, approved the present study [No.2020-02]. Written informed consent was obtained from the study participants or his/her guardians for clinic examinations, genetic tests and publication of this case including any potentially identifiable images.

Patients and clinical evaluation

The proband, a 9-year-old girl, and her mother and elder brother from a two-generation Chinese family were affected by congenital cataract. The detailed medical histories of the patients were noted. Ophthalmic examinations (visual acuity, refraction, colour vision, slit lamp, intraocular pressure (IOP) and fundus photography) were offered to the patients.

Genomic DNA extraction

Peripheral venous blood (5 mL) was collected in BD Vacutainers (BD, San Jose, USA) containing EDTA for genomic DNA isolation. Genomic DNA was extracted by using TIANamp Blood DNA Kit (#DP348-03, TIANGEN BIOTECH, Beijing, China). The purity and concentration of prepared DNA were determined by using NanoDrop 1000 (Thermo Fisher, USA). Qualified samples (concentration ≥ 20 ng/μl, A260/280 = 1.8 ~ 2.0, total DNA ≥3 µg) were sent for sequencing.

Whole exome sequencing (WES)

WES was conducted at Yikon Genomics, Inc (Suzhou, China). Briefly 3 µg of genomic DNA was used for library preparation. The library was captured using the xGen Exome Research Panel v1.0 (Integrated DNA Technologies, Inc.,USA), and 2 x 76 bp paired-end reads were generated on a HiSeq2000 (Illumina, San Diego, USA). Reads that did not pass Illumina’s standard filters were removed prior to alignment. Then, the remaining reads were aligned to the reference UCSC Genome human genome (hg19), using Burrow-Wheeler Aligner (BWA) (v. 0.5.9) software. The Haplotype Caller tool of Genome Analysis Toolkit (GATK) was used for single nucleotide variation, local realignment around indels (short insertions and deletions), and for coverage assessment. A custom filtering protocol was applied to the whole exome data: (a) variants with mapping qualities < 30; (b) the total mapping quality zero reads < 4; (c) approximate read depth < 5; (d) query quality (QUAL)< 50.0; and (e) phredscaled p-value using Fisher’s exact test to detect strand bias > 10.0. The public databases including 1,000 genomes, dbSNP, HGMD and gnomAD, as well as a local database at Yikon Genomics, Inc. were applied for variants annotation. Sanger sequencing was performed to verify the identified variants in the proband and affected individuals in the family. The sequencing data are uploaded and stored in the European Molecular Biology Laboratory (EMBL).

In silico analysis

Online bioinformatic tools Sorting Intolerant from Tolerant (SIFT) and Protein Variation Effect Analyzer (PROVEAN) were used to predict the impact of variation on protein function. A SIFT score < 0.05 predicted to be damaging (D). A PROVEAN score equaling to or below −2.5 was considered deleterious (D), whereas a score above −2.5 was considered neutral (N).

## Results

Clinical data

**Figure 1:**
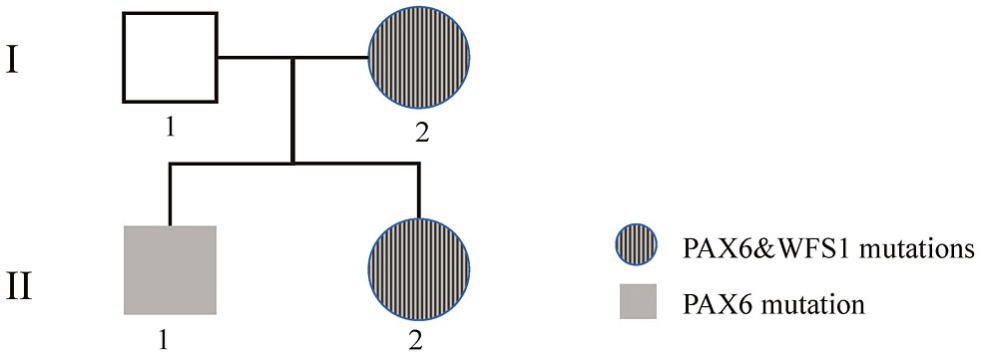
Pedigree of the family

**Figure 2:**
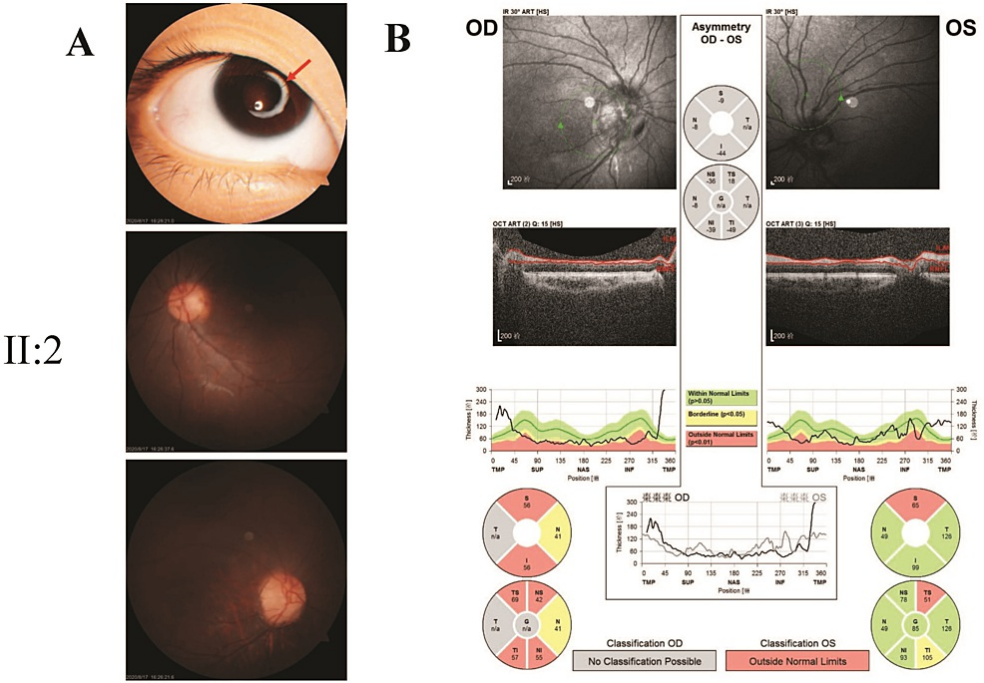
Ocular examination of the proband (Patient II-2) A: Slit-lamp aspects of the ocular anterior segment as well as of the lens and funduscopy photographs. The red arrow points to remnants of the lens capsule and the zonular fibers after lensectomy; B: Optical coherence tomography (OCT) analysis of optic nerve fiber.

**Figure 3:**
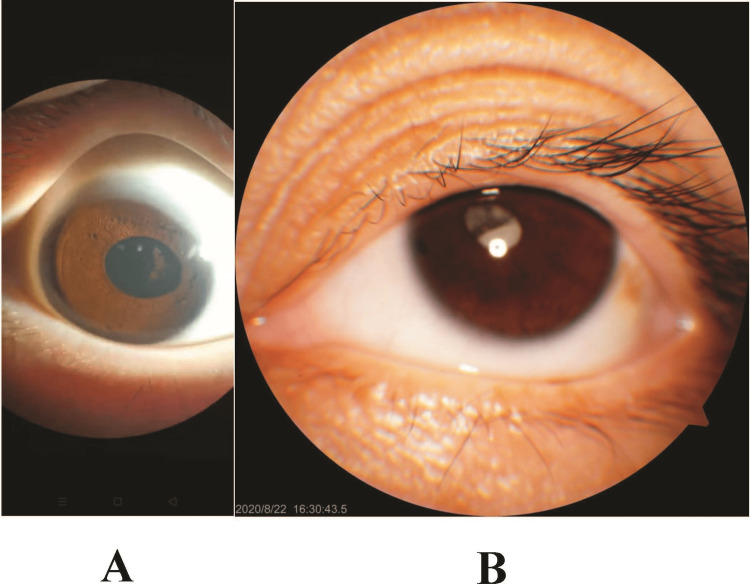
Lamp aspects of the ocular anterior segment as well as of the lens from (A) the boy (Patient II-1) and (B) the mother (Patient I-2).

The proband (Patient II-2, Figure 1) received lensectomy due to blindness at the age of two that resulted from bilateral congenital cataract. Ophthalmologic examinations identified aphakic eye after surgery and nystagmus (Figure 2A). Visual acuity were 0.13 OD and 0.2 OS. Cornea was clear without vascularization and IOP was in normal range. Optical coherence tomography (OCT) showed that the optic nerve fibers were slightly thinner than the normal range, and especially in the upper and lower quadrants. The results of temporal side analysis could not be obtained due to nystagmus (Figure 2B). The proband’s elder brother (Patient II-1, Figure 1) solely also suffered from obvious bilateral nuclear cataracts, with a better visual acuity of 0.06 OD and 0.08 OS (Figure 3A). The proband’s mother lost vision in her left eye at the age of 20, and extracapsular cataract extraction surgery was performed without intraocular lens implant. The mother's right eye lost almost all vision in her 30s, now having only light perception. Nuclear cataracts with bilateral nystagmus were observed for the mother (Figure 3B). The pupils of the patients did not reside in the center of the optic axis, inclining to the nasal side when not dilated. Fundus photography showed normal results in all the patients. Family history of eye diseases in the generation of grandparents was denied. No past histories of diabetes, hearing impairment, or neurological disorders were reported in any member of the two-generation Chinese family.

Genetic findings

**Figure 4:**
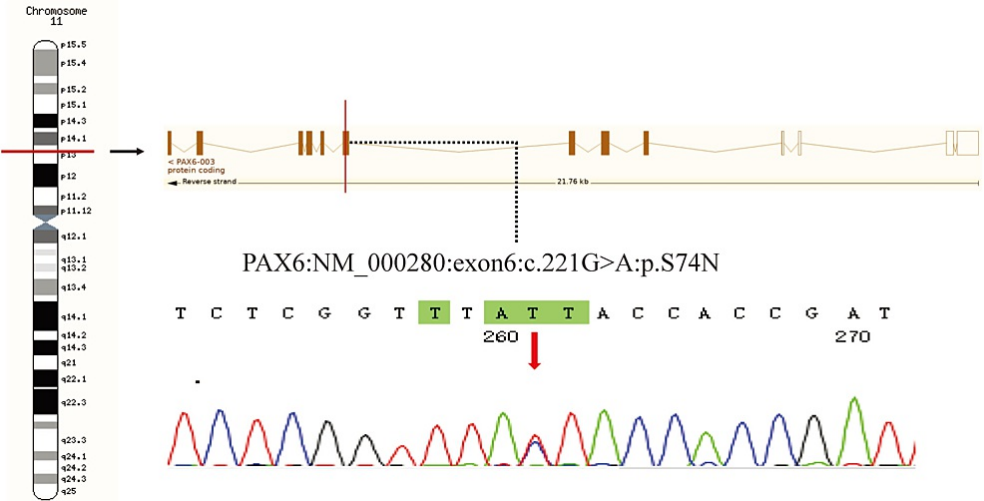
Sequence analysis and identification of the novel mutation of PAX-6 in the affected Chinese family with congenital cataracts

**Figure 5:**
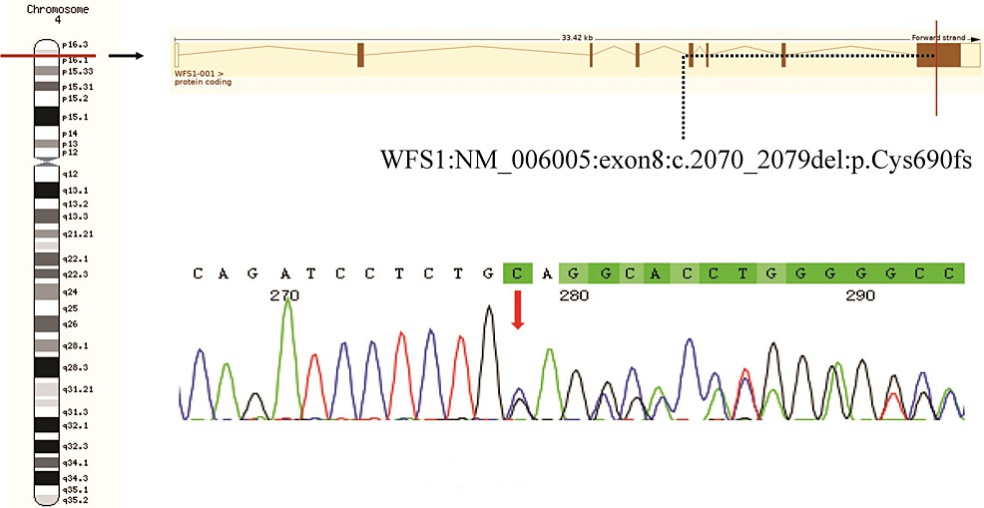
Sequence analysis and identification of the novel mutation of WFS1 in the affected Chinese family with congenital cataracts

WES identified heterozygous mutations of PAX6 (NM_000280: exon6: c.221G>A: p.Ser74Asn) on chromosome 4 (Figure 4) and WFS1 (NM_006005:exon8: c.2070_2079del: p.Cys690TrpfsTer17) on chromosome 11 (Figure 5) in the proband. Sanger sequencing confirmed that both the proband and her mother (Patient I-2) are carriers of the mutations. The proband’s elder brother (Patient II-1) inherited the PAX6 c.221G>A mutation only from the mother. These two variations were not present in the healthy father (I-1) and two unrelated healthy controls.

Loss of function of WFS1 is responsible for autosomal recessive Wolfram syndrome type 1. The full-length WFS1 protein consists of 890 amino acid residues. The c.2070_2079del variation is present on biologically relevant transcript and predicted to cause framshift and pre-termination of translation, yielding a truncated protein (p.Cys690TrpfsTer17) that losses the putative topological domain (amino acid residues 653-869) and trans-membrane domain (amino acid residues 870-890) in the C-terminal and is likely to be loss-of-function. This variation was absent from East Asians in gnomAD and literature. According to the American College of Medical Genetics and Genomics (ACMG) guidelines, the WSF1 c.2070_2079del variant was classified as likely pathogenic.

PAX6 mutations can lead to a group of heterogeneous congenital eye diseases of autosomal dominant inheritance. The PAX6 c.221G>A (p.Ser74Asn) variation has not been reported for East Asians in gnomAD. The substitution of serine 74 by asparagine in PAX6 protein scored -2.51 and 0.00 in PROVEAN and SIFT, respectively, indicating that this mutation is deleterious and damaging (Table 1). According to the ACMG guidelines, the PAX6 c.221G>A variation was considered as variation of uncertain significance.

**Table 1:** In silico analysis of the mutations in PAX6 and WFS1 #SEQ: Number of sequences used for prediction; #CLUSTER: Number of clusters used for prediction

VARIATION	PROTEIN SEQUENCE CHANGE						PROVEAN PREDICTION				SIFT PREDICTION		
INPUT	TYPE	PROTEIN_ID	POSITION	RESIDUE_REF	RESIDUE_ALT	SCORE	cutoff=-2.5	#SEQ	#CLUSTER	SCORE	cutoff=0.05	MEDIAN_INFO	#SEQ
PAX6 ENSP00000368418,74,S,N	Missense	ENSP00000368418	74	S	N	-2.51	Deleterious	196	30	0.000	Damaging	2.81	290
wfs1 4,6303591,GCAGCCACCTG,G	Frame shift	ENSP00000226760					NA	87	29	NA	NA	NA	NA

## Discussion

Based on the genetic analysis of the Chinese pedigree, we have identified novel heterozygous mutations of PAX6 c.221G>A and WFS1 c.2070_2079del that are associated with severe congenital cataract even causing blindness in childhood with nystagmus, while the monogenic PAX6 c.221G>A mutation alone can also cause obvious bilateral congenital cataract.

Cataracts are defined as a loss of lens transparency, and the formation of insoluble opaque protein aggregates is the disease hallmark [10,11]. PAX6 plays a fundamental role in lens development in coordination with SRY-Box Transcription Factor 2 (SOX2) via inducing the surface ectoderm to form the lens placode, which invaginates and forms the lens vesicle at early stage of gestation [12,13]. In adulthood, PAX6 is mostly expressed in cornea, iris, and lens [14]. Deficiency in PAX6 gene can result in a wide range of congenital eye anomalies, among which the most common are aniridia, nystagmus, and foveal hypoplasia, with cataracts being usually reported as an accompanied disorder. Few studies have ever reported the association of PAX6 with isolated congenital cataract. Moreover, common mutations associated with cataracts of various morphologies include genes encoding crystallins (CRYA, CRYB, and CRYG), lens specific connexins (Cx43, Cx46, and Cx50), major intrinsic protein (MIP) or aquaporin, heat shock transcription factor 4 (HSF4), etc. but PAX6 is neglected [15-22]. The present study addresses of a juvenile patient who carried a heterozygous PAX6 c.221G>A (p.Ser74Asn) variation solely caused non-syndromic congenital cataract. This case provides new insight into the phenotype spectrum associated with PAX6 mutations. In search of literature, we also noticed the documentation of PAX6 c.220A>T (p.Ser74Cys) variant that has been linked with aniridia in the Clinvar database (Accession: VCA000578328.1). It would be interesting to uncover the molecular mechanisms that underlying distinct phenotypes regarding substitution of the highly conserved serine-74 residue by different amino acids in PAX6 protein.

Wolframin, the encoding protein of the WFS1 gene acts to regulate calcium homeostasis in cells. Mutations in WFS1 are well known to cause autosomal-recessive Wolfram syndrome, a severe neurodegenerative disease mainly characterized by juvenile-onset diabetes mellitus and optic atrophy (OMIM 222300) [7]. However, the variants of WFS1 associated with non-syndromic congenital cataract have also been described. Berry et al. identified a c.1385A>G (p.Glu462Gly) variant that causes autosomal dominant congenital nuclear cataract in humans [8]. Wang et al. reported that the mutation c.1235T>C (p.Val412Ala) in WFS1 may be associated with the development of autosomal dominant congenital cataract [23]. Therefore, there is a trend that the majority of recessive inactivating mutations of WFS1 cause typical Wolfram syndrome, whereas dominant non-inactivating mutations of WFS1 are associated with less severe but more heterogeneous phenotypic manifestations [8,24]. In this study, we observed that the heterozygous mutations of PAX6 (c.220A>T/p.Ser74Cys) and WFS1 (c.2070_2079del/p.Cys690TrpfsTer17) were associated with more severe congenital cataracts than the monogenic heterozygous mutation of PAX6 (c.220A>T/p.Ser74Cys) in a Chinese family. Though the WFS1 c.2070_2079del variant is speculated to yield a loss-of-function mutant, at this moment, we are unable to specify the genetic contribution of WFS1 on cataracts. Further mechanistic investigations are warranted to reveal the interplay between PAX6 and WFS1 in the pathogenesis of congenital cataracts.

Our proband had cataract surgery for her eyes when cataract was diagnosed, and intraocular lens were implanted. After the surgery, her visual acuity returned to 0.15 OD and 0.15 OS. The mother’s right eye was treated with phacoemulsification and intraocular lens implantation, and the visual acuity has returned to 0.1.

## Conclusions

PAX6 c.221G>A and WFS1 c.2070_2079del were novel heterozygous mutations that are associated with severe congenital cataract even causing blindness in childhood with nystagmus. The PAX6 mutation (c.221G>A) can cause congenital cataract, and the WFS1 mutation (c.2070_2079del) may interactively aggravates this process. These findings may increase our understanding of the genetic etiology of congenital cataract. Further mechanistic investigations are warranted to reveal the interplay between PAX6 and WFS1 in the pathogenesis of congenital cataracts.

## Appendices

The supplementary data is on https://github.com/hao203/journal-file/tree/main/cataract. The sequencing data are uploaded and stored in the EMBL (https://ega-archive.org/datasets/EGAD00001008267).

WES report.pdf: WES report of the proband

Slit lamp of the boy.mp4: The video of slit lamp examination of the boy

Sanger sequencing report.pdf: Sanger sequencing report of the mother and the boy

The father.pdf: Sanger sequencing report of the healthy family member (the father)
